# The association between pelvic girdle pain and sick leave during pregnancy; a retrospective study of a Norwegian population

**DOI:** 10.1186/s12884-015-0667-0

**Published:** 2015-10-05

**Authors:** Stefan Malmqvist, Inger Kjaermann, Knut Andersen, Inger Økland, Jan Petter Larsen, Kolbjørn Brønnick

**Affiliations:** Department of Health Studies, University of Stavanger, Stavanger, Norway; Norwegian Centre for Movement Disorders, Stavanger University Hospital, Stavanger, Norway; Private practice, Kristiansand, Norway; Department of Obstetrics and Gynaecology, Stavanger University Hospital, Stavanger, Norway; The Norwegian Centre for Movement Disorders, Stavanger University Hospital, Stavanger, Norway; Network for Medical Sciences, University of Stavanger, Stavanger, Norway

**Keywords:** Pelvic girdle pain, Sick leave, Pregnancy, Coping

## Abstract

**Background:**

The incidence of pelvic girdle pain (PGP) in pregnancy is wide ranged depending on definition, the utilised diagnostic means, and the design of the studies. PGP during pregnancy has negative effects on activities of daily living and causes long sick leave, which makes it a major public health issue. Our objectives were to explore the frequency of sick leave in pregnancy due to PGP, assess the relationship between different types of pain-related activities of daily living, examine physical workload, type of work in relation to sick leave, and to explore factors that make women less likely to take sick leave for PGP.

**Methods:**

All women giving birth at the maternity ward of Stavanger University Hospital, Norway, were asked to participate and complete a questionnaire on demographic features, PGP, pain-related activities of daily living, sick leave in general and for PGP, frequency of exercising before and during pregnancy. Drawings of pelvic girdle and low back area were used for the localization of pain. PGP intensity was then rated retrospectively on a numerical rating scale. Non-parametric tests, multinomial logistic regression and sequential linear regression analysis were used in the statistical analysis.

**Results:**

PGP is a frequent and major cause of sick leave during pregnancy among Norwegian women, which is also reflected in activities of daily living as measured with scores on all Oswestry disability index items. In the multivariate analysis of factors related to sick leave and PGP we found that work satisfaction, problems with lifting and sleeping, and pain intensity were risk factors for sick leave. In addition, women with longer education, higher work satisfaction and fewer problems with sitting, walking and standing, were less likely to take sick leave in pregnancy, despite the same pain intensity as women being on sick leave.

**Conclusions:**

A coping factor in pregnant women with PGP was discovered, most likely dependant on education, associated with work situation and/or work posture, which decreases sick leave. We recommend these issues to be further examined in a prospective longitudinal study since it may have important implications for sick leave frequency during pregnancy.

## Background

Pelvic girdle pain (PGP) in pregnancy is an unclear and poorly understood condition. There is no official acknowledged nomenclature, hence an abundance of different terminologies have been used in studies to describe it [1, 2]. The European guidelines for the diagnosis and treatment of PGP describe the localization of pain as“… experienced between the posterior iliac crest and the gluteal fold, particularly in the vicinity of the sacroiliac joints (SIJ). The pain may radiate in the posterior thigh and can also occur in conjunction with/or separately in the symphysis” [3].

The incidence of pain in the pelvic girdle region in pregnancy ranges from 4 to 76 % depending on the definition, the utilised diagnostic means, and the design of the studies [4]. One review study concludes that approximately 45 % of all pregnant women suffer from lumbopelvic pain in pregnancy, and approximately 20 % of these have isolated PGP [1].

PGP accounts for 37–72 % of sick-leave in pregnancy and length of the sick leave had been found to be 12–15 weeks on average [4–8]. Other pregnancy complications may also occur in women with PGP, leading to sick leave [7]. However, women with a high degree of self-reported PGP have longer sick-leave duration than others, and these pain symptoms were in one study reported to bring about 80 % of sick leaves during pregnancy. The authors argued that this makes PGP during pregnancy a major public health issue [7].

Several risk factors for developing PGP during pregnancy have been identified, such as work load, previous PGP, and previous trauma to the pelvis [1, 3, 7–10]. PGP during pregnancy also has negative effects on activities of daily living (ADL) [7–11]. On average, these women do not experience continuous PGP, but activities of daily living such as walking, standing, sitting, lying down, or changing positions may become painful after some time. There is commonly a difficulty in walking fast or over long distances [1]. Disabling problems among women with lumbopelvic pain during pregnancy range from 21 to 81 % (median 28 %) [1]. A moderate to high Oswestry Disability Index (ODI) in women with pelvic pain during pregnancy has been identified as a risk factor for persistent PGP 3–6 months after birth [12].

The objectives of this study were to explore the frequency of sick leave in pregnancy due to PGP and to assess the relationship of different types of pain-related ADLs, physical workload, and type of work with sick leave due to PGP. Further, we wanted to explore factors that induce less sick leave due to PGP, by contrasting women who were matched for PGP, but differed by having been versus not having been on sick leave for PGP. The final objective was to explore the relative contribution of PGP to total amount of sick leave in pregnancy.

## Methods

The data used in this study were collected in the period March – June 2009 at the maternity ward of Stavanger University Hospital, Norway [13]. All women giving birth at the hospital during this period were asked to participate and to fill in a study questionnaire. Inclusion criteria were a term singleton pregnancy of at least 36 weeks and good competence in the Norwegian language. The hospital has the only birth department in the southern part of the county of Rogaland, with a population of about 330,000 inhabitants.

Within 24 h after the delivery, the women received both oral and written information about the study from a midwife. To obtain inclusion of an unselected sample, all women were encouraged to give their informed consent to participate. The study was carried out in accordance with the Helsinki Declaration II and was approved by the Regional Ethics Committee of Western Norway.

To assess if the study population was a representative sample of the delivering women, we compared demographic and clinical characteristics of the study population with that of the general delivery database at the hospital and found a nearly complete match [13]_._

The women completed a questionnaire on demographic features, pain in pelvic girdle area, pain-related ADL, sick leave in general and due to PGP, and frequency of exercising before and during pregnancy. The questionnaire was produced and specially designed by the research group, based on previous studies and the experience of the team [13].

The women marked location of pain on drawings of the pelvic girdle and low back included in the questionnaire package. The pelvic girdle and the low back were labelled, and separated according to boundaries described in the European guidelines for the diagnosis and treatment of PGP [3]. Pain intensity for PGP was then rated retrospectively on a numerical rating scale (NRS) from 0 to 100, for each month of the pregnancy, in order to collect information on appearance of symptoms and peak intensity pain during pregnancy. In this study the score 0 meant “No pain” and 100 “Unbearable pain”, and average pain intensity for PGP was calculated from the values reported in all months.

Information on pain-related ADL was collected through the Oswestry Disability Index (ODI), which is one of the principal condition-specific outcome measures for defining disabling effects from spinal disorders and pelvic girdle pain [3, 14, 15]_._

The questionnaires also provided information on number of years of education, level of physical work load (a five level scale running from very light to very heavy), type of work (in free text but coded into mainly seated, mainly standing, mainly mobile), work satisfaction (a five level scale running from very bad to very good), and height and weight before pregnancy and weight at delivery. Further variables included number of previous births, exercising habits before pregnancy, defined as regular exercise at least twice weekly (yes or no). Body mass index (BMI) was calculated as weight/length^2^.

In Norway the employer is obliged to pay sick pay for the first 16 calendar days (employer's period) [16]. After that, the Norwegian Labour and Welfare Service (NAV) will take over the responsibility. The employee is required to notify the employer as soon as possible of any absence due to illness. The duty to report includes only information regarding the absence. The obligation to pay sick pay commences from the day the employer is notified about the absence, unless the employee has been prevented from notifying the employer immediately.

In the present study sick leave was assessed in two different ways. First, the women were asked about the total number of weeks of full-time sick leave in the pregnancy, as well as the total number of weeks with part-time sick leave and sick leave percentage. Weeks of sick leave in total were calculated by adding the full-time sick leave weeks to the part time weeks adjusted for sick leave percentage. After reporting the total amount of sick leave, the women were asked to indicate a primary cause of their sick leave. Second, in association with the section of the questionnaire concerning NRS of pain during the pregnancy, the women were also asked whether they had been on sick leave due to PGP in any month of the pregnancy and to indicate when. It was not possible to determine the number of consecutive weeks of 100 % sick leave due to any specific cause from the available information. For instance, several women only reported “pain” without any specific details as the main cause of sick-leave in pregnancy. To determine whether the women had sick leave due to PGP, we combined the available information. If the women reported any sick leave due to PGP in any month of the pregnancy, they were classified as having sick leave due to PGP. Women, who explicitly stated that PGP was the primary cause of their sick leave, but who did not indicate sick leave due to PGP in any specific month of pregnancy in the NRS-section of the questionnaire, were also classified as having sick leave due to PGP. This procedure resulted in three groups: women with no reported sick leave, women with sick leave but without any indication of PGP being the cause, and finally, women who had sick leave and reported PGP as the cause of, at least, parts of their sick leave.

Descriptive data are presented as mean values and standard deviations for continuous variables and as frequencies for categorical variables. For comparisons between groups the non-parametric Kruskal-Wallis statistics were used, applying Bonferroni correction. Pairwise follow-ups were performed with the group who had sick leave due to PGP as reference, whenever significant omnibus group differences were found. For categorical data, chi-square statistics were computed, and 2 × 2 table follow-ups were used for pairwise comparisons between the group with sick-leave due to PGP vs the other groups.

Multinomial logistic regression analysis was used to investigate the independent contribution of variables hypothesized to affect whether the women had been on sick leave due to PGP. Forced entry was used with age, education, parity, and average PGP in order to adjust for these variables in the final model. As an exploratory approach, single items from ODI (except sex and pain intensity) were entered in a stepwise procedure together with workload, work satisfaction and seated work, using a likelihood ratio based criterion with *p* < .05 for entry and *p* < .10 for removal.

In order to explore factors associated with the total amount of sick leave in pregnancy, a sequential linear regression analysis was conducted, using total number of calculated weeks of sick leave (weeks of 100 % sick-leave + weeks of part-time sick leave multiplied by sick leave percentage) for any reason as dependent variable. In the first block, the grand average of monthly reported PGP was entered, in order to analyse the unadjusted effect of PGP on weeks of sick leave. In block 2, all relevant ODI items were entered using a stepwise procedure (*p* < .05 to enter, *p* < .01 to exclude a variable). In block 3, years of education, pre-pregnant BMI, workload, age, standing work and mobile work were entered, using the same type of stepwise procedure as in block 2. Finally, in block 4, work satisfaction and depression in pregnancy were entered, also with a stepwise procedure. Thus, only block 1 included a forced entry variable, average PGP, as we wanted to explore unadjusted and adjusted effects of PGP on weeks of sick leave.

In order to investigate factors that may decrease the effect of PGP on sick leave, we identified all women with PGP who did not have any sick leave in pregnancy. A macro written in Microsoft Excel (Visual Basic) then chose a random woman having been on sick leave, and who matched the average PGP score of a woman without sick leave. If a perfect match was not found, a difference of +/−1 point on the PGP score was accepted. If still no match was found, the subject was discarded. Hence, this procedure chose two equivalent groups with regard to average PGP, but with and without sick leave. We then compared these groups on the same variables as for the sick leave due to PGP, vs no sick leave, vs sick leave due to other reasons groups.

Effect sizes were reported as standardized mean differences (Cohen’s D), using Bonferroni correction, which can be interpreted as small (around 0.3) medium (around 0.5) and large (0.8 to infinity) [17].

All analyses were performed using SPSS 21 (IBM, New York, NY), and results were considered significant at *p* < .05.

## Results

### Study population

In all, 1204 women who gave birth at Stavanger University Hospital during the inclusion period were invited to participate in the study. After exclusions, there were 994 women eligible for the study. In addition, 336 women did not return a questionnaire and 89 did not complete a received questionnaire. One woman did not report having a job or profession, nor any workload, and was excluded from the analyses. The final study population thus consisted of 568 women. Of these, 165 (29 %) reported that they had experienced isolated PGP during the pregnancy.

The sample’s demographic data and descriptive statistics for the variables used in the multivariate analyses are shown in Table 1. Several significant differences are shown between subjects who reported sick leave due to PGP vs. those who did not [Table 1]. In Table 2 we compare the group with sick leave due to PGP with the no sick leave group and the group with sick leave due to other causes on single items from the ODI. We report effect sizes to enable a direct comparison using a standardized scale [Table 2].

**Table 1:** Descriptive statistics

	Sick leave for PGP	No sick leave	Sick leave for other reason	p****	Total
N (%)	193 (34 %)	139 (24 %)	236 (42 %)		568 (100 %)
Age	29.7 (4.3)	30.5 (4.8)	29.8 (5.0)	=.254	30.0 (4.7)
Education	14.5 (2.4)	15.7 (2.4)*	15.4 (2.6)*	<.001	15.1 (2.6)
BMI before pregnancy	24.8 (4.6)	23.1 (3.6)*	23.4 (4.2)*	<.001	23.8 (4.3)
Total sick leave weeks	10.8 (9.4)	0.0 (0.0)*	8.4 (8.9)*	<.001	7.2 (9.0)
Workload	3.0 (1.1)	2.4 (1.1)*	2.6 (1.2)*	<.001	2.6 (1.1)
Work satisfaction	4.4 (0.8)	4.6 (0.7)*	4.3 (0.9)	<.001	4.4 (0.8)
Average PGP	26.8 (15.1)	6.7 (10.4)*	6.1 (10.0)*	<.001	13.3 (15.5)
Average LBP	13.2 (16.9)	4.7 (9.1)*	6.6 (11.4)*	<.001	8.4 (13.4)
Pain-related ADL (ODI)	1.9 (0.8)	0.9 (0.9)*	1.0 (0.8)*	<.001	1.4 (0.9)
Depressed	1.4 (0.5)	1.3 (0.5)	1.4 (0.6)	=.055	1.4 (0.5)
Mean no. of previous births	1.00 (0.06)	0.94 (0.09)	0.79 (0.05)***	<.05	0.90 (1.00)
Regular exercise before pregnancy	63 (33 %)	67 (49 %)**	94 (40 %)	=.013	224 (39 %)
Seated work	51 (27 %)	68 (49 %)*	81 (34 %)	<.001	200 (35 %)

*PGP* Pelvic girdle pain, *LBP* Low back pain. Pairwise comparison with sick-leave for PGP: *p < .001, **p < .01, ***p < .05

****Kruskal-Wallis omnibus test. Bonferroni-corrected alpha = 0.0038

**Table 2:** Oswestry disability index items

	Sick leave for PGP	No sick leave		Sick leave for other reason		Total
	*N* = 190	*N* = 96		*N* = 154		*N* = 440
ODI item	Mean (SD)	Mean (SD)	E.S.^a^	Mean (SD)	E.S.^a^	Mean (SD)
Pain intensity	2.76 (0.86)	1.67 (1.13)	1.142	1.81 (0.99)	1.024	2.19 (1.09)
Personal care	1.23 (1.40)	0.59 (0.97)	0.591	0.53 (0.98)	0.655	0.85 (1.10)
Lifting	2.18 (1.19)	0.95 (1.12)	1.056	1.30 (1.16)	0.751	1.60 (1.27)
Walking	1.63 (0.99)	0.65 (0.94)	1.007	0.85 (1.05)	0.762	1.15 (1.09)
Sitting	1.68 (0.96)	0.80 (0.98)	0.905	1.07 (1.11)	0.589	1.28 (1.08)
Standing	2.44 (1.24)	1.17 (1.28)	1.018	1.48 (1.27)	0.769	1.83 (1.37)
Sleeping	1.67 (1.02)	0.82 (0.88)	0.872	1.04 (0.88)	0.657	1.26 (1.01)
Sex	1.75 (1.50)	0.76 (1.19)	0.707	0.76 (1.30)	0.701	1.18 (1.45)
Social function	1.89 (1.26)	0.83 (1.17)	0.862	0.79 (1.14)	0.911	1.27 (1.31)
Travelling	1.63 (1.26)	0.61 (1.00)	0.860	0.82 (1.11)	0.680	1.12 (1.24)

E.S.: Effect size (Cohen’s d. >0.8 is considered large)

^a^All differences of means statistically significant assuming a Bonferroni corrected alpha of *p* < .005

All ODI-items were significantly higher in the group who had been on sick leave for PGP than in both the other groups. The effect sizes were all moderate to large (Cohen’s d > 0.6).

### Factors associated with sick leave due to PGP

Individual risk factors with odds-ratios and confidence limits resulting from the multinomial regression analysis are shown in Table 3.

**Table 3:** Multinomial regression with sick leave due to PGP as reference category

	*P*	Odds ratio	C.L. Low	C.L. High
No sick-leave				
Age	.157	1.056	.979	1.138
Education	.074	1.113	.990	1.252
Pelvic pain	.001	.955	.930	.981
No. of previous births	.667	.915	.612	1.369
ODI: Lifting	.011	.622	.432	.895
ODI: Sleep	.008	.521	.321	.846
ODI: Social life	.294	1.206	.850	1.713
Work Satisfaction	.049	1.607	1.001	2.580
Sick-leave due to other reason				
Age	.129	1.051	.985	1.122
Education	.262	1.054	.961	1.157
Pelvic pain	.000	.951	.932	.971
No. of previous births	.128	.760	.533	1.083
ODI: Lifting	.020	.708	.530	.946
ODI: Sleep	.622	.916	.646	1.299
ODI: Social life	.105	.785	.586	1.052
Work Satisfaction	.814	.960	.681	1.352

C.L.: 95 % confidence limits

All results in Table 3 refer to the group with sick leave due to PGP as reference category. The estimated pseudo R^2^ was quite high (Nagelkerke R^2^ = .40) and the total correct classification percentage was 62 %. We see that work satisfaction, as well as lower scores for ODI-lifting, sleep and average pain intensity, significantly predicted affiliation to the no sick leave group. The group with sick leave due to other reasons had lower score on average pain intensity and ODI-lifting [Table 3].

### Coping with the effects of pelvic girdle pain

The matching procedure resulted in two groups with 50 subjects in each group. The group with sick leave due to PGP and the group with no sick leave (coping group) had similar PGP intensities of approximately 18. Univariate Mann–Whitney *U* test revealed that the coping group on average had longer education (15.8 vs 14.8 years), *p* = .022 and higher work-satisfaction (4.66 vs 4.32), *p* = .014. Finally, the scores on several ODI-items were lower in the coping group [Table 4].

**Table 4:** ODI in women who had PGP, with and without sick leave for PGP

ODI item	No sick-leave for PGP	Sick-leave for PGP	E.S.	*p*
Pain intensity	2.30 (0.84)	2.33 (0.88)	0.039	=.954
Personal care	0.90 (1.11)	0.94 (1.14)	0.033	=.889
Lifting	1.40 (1.21)	1.90 (1.29)	0.395	=.044
Walking	0.96 (1.01)	1.44 (0.97)	0.483	=.011
Sitting	0.96 (1.00)	1.46 (0.87)	0.528	=.003
Standing	1.50 (1.33)	2.00 (1.22)	0.392	=.031
Sleeping	1.12 (0.85)	1.48 (1.03)	0.379	=.113
Sex	1.18 (1.41)	1.23 (1.49)	0.037	=.969
Social function	1.26 (1.27)	1.33 (1.31)	0.056	=.677
Travelling	0.92 (1.12)	1.25 (1.23)	0.280	=.180

*ES* Effect size (Cohen’s d); Bonferroni-corrected alpha = 0.005

These results differ from the ODI-results in Table 2, as the effect sizes between the groups are very different for the different items. If a strict Bonferroni-correction is applied, only the ODI score for sitting is higher in the group with sick leave due to PGP (*p* < .003). However, also walking and standing differ if uncorrected *p*-values are applied.

## Discussion

This study shows that PGP is frequent and a major cause of sick leave during pregnancy among Norwegian women, which is also reflected in problems with ADL as measured with scores on all ODI items. In the multivariate analysis of factors related to sick leave and PGP we found that work satisfaction, problems with lifting and sleeping, and pain intensity predicted sick leave. In addition, we found that women with longer education, higher work satisfaction and less problems with sitting, walking and standing, were less likely to take sick leave during pregnancy, despite having the same pain intensity as women being on sick leave. These findings may have implications for planning of measures that could reduce sick leave among pregnant women.

Most studies describe percentages of pregnant populations on sick leave, and mainly use lumbopelvic pain as a general term, describing the location of pain. Few specify length of sick leave or differentiate PGP from low back pain for a more specific location of pain. Also, the majority of studies are prospective or cross-sectional. Our study was retrospective and shows a sick leave percentage and span similar to other studies with the same methodology. In our study, 34 % of the women had been on a median of eleven weeks sick leave for PGP during the pregnancy. In a Swedish retrospective study, sick leave for pain in the pelvic girdle area was reported among 48 % of the pregnant women, with a mean span of sick leave of 12 weeks [11]. In another retrospective study, 41 % had been on sick leave for PGP, but duration was not described [18]. Taken together, our and previous studies underline the importance of PGP as a major cause of sick leave during pregnancy.

A previous Norwegian study showed that sick leave increases for each trimester in pregnancy. It revealed that fatigue and sleep problems seem to be the main risk factor for sick leave, followed by nausea, vomiting, exercising less than weekly, chronic PGP before or during pregnancy, conflicts in the workplace, and lower education [18]. In contrast, Mogren in a retrospective study found lumbopelvic pain to be the main cause of sick leave during pregnancy [11]. This finding is supported by our study, and Robinson and co-workers who showed that almost a third of all delivering women were sick listed due to PGP during pregnancy [18]. A result confirming that PGP accounts for a great part of sick leave during pregnancy.

Our study shows that ADL were significantly more difficult to carry out for pregnant women on sick leave for PGP than for women on sick leave for other causes. We found independent significant risk factors to be lower education, heavy workload and low work satisfaction. This association has previously been shown in three other studies in which pregnant women with demanding working conditions presented increased incidence of PGP in pregnancy, while those with opportunity to adjust their work pace reported a better health status than women without this possibility [19–21]. Risk indicators for long-term sick leave during pregnancy have been shown to be less qualified work and heavy work load [22]. Lower education level has also been found to associate with higher pain intensity during pregnancy [23].

The ODI score on sitting scored the highest significant effect size in the group with sick-leave due to PGP (*p* < .003). It is known that prolonged sitting may alter the passive stiffness of the lumbar spine. Erector spinae muscles fatigue induces a shift in load-sharing towards passive stabilizing structures. Loss of muscle contribution together with or without laxity in the viscoelastic tissues may have a substantial impact on post fatigue stability [24].

In our study, a matching procedure revealed that the group with sick leave due to PGP had a lower PGP intensity score than the group with no sick leave. The reason for this unexpected find may be that pain is a complex construct that contributes to profound physical and psychological dysfunction, and the experience of it is modulated by physical and psychological factors [25]. Following the bio-psychosocial model, emotional distress may predispose people to experience pain or may be a moderator that amplifies or inhibits the severity of pain [26]. Traumatic experiences related to pregnancy seem to be associated with lumbopelvic pain and physical ability up to 6 months after delivery [27].

Certain individuals can stand the pain, have less catastrophizing tendencies, show more positive social responses to pain, and more organized health care and medication patterns. These coping skills are displayed as effective functioning despite exposure to stressful circumstances and/or internal distress [28]. Most important psychological contributors to individual well-being and coping stress responses are positive emotions, which appear to buffer individual reactivity to pain [25].

In a retrospective study, with a similar design to ours, the authors found that pregnancy seems to be a period during which a sick-listed is prone to be influenced by attitudes and coping strategies in order to achieve needed rest prior to delivery [29]. They concluded that certain social conditions and attitudes are likely to explain why pregnant women are on sick leave [29]. Chang and co-workers suggest that education and interventions targeting passive coping and stimulating resilience may be useful to prevent PGP during pregnancy turning chronic [23]. In Korea, a back-pain-reducing program was effective in reducing the intensity of back pain experienced by pregnant women [30].

Our findings show that women may benefit from a pre-pregnancy and pregnancy strategy program decreasing the risk for pregnant women to end up on sick leave for PGP. According to our results the strategy should contain information about the value of pre-pregnancy regular physical exercise in order to withstand physical workload during pregnancy. In order to boost coping towards PGP and sick leave, information about physical, physiological and psychological changes and challenges in pregnancy, should be included. Employers should through an incentive be encouraged to ergonomically adapt the pregnant employees’ workplace in order to decrease work load and thus maintain, or even improve, work satisfaction.

There are limitations in the present study. We introduced several tactics to obtain a high awareness of the importance of the study, both among the women and assisting midwives, with the objective to enrol all women who fulfilled the inclusion criteria during a given time period. Nevertheless, our response rate was rather low, in spite of aforementioned initiatives, but we did find that age and parity of the studied population was identical to the general population of women that gives birth at the hospital. We therefore believe the results from this study to be representative for pregnant women in the study area, and most probably also in the rest of Norway.

Ideally, to use the term PGP a physical examination is required, but it could not be done in this study. Another limitation is that all our data are based on questionnaires with retrospective data collection. We are aware that recalling pain and disability experienced long time ago is considered an unreliable way to collect information. Regarding the occurrence and duration of sickness absence during pregnancy has the agreement between self-report and a public registry been investigated [31]. Mainly because of low precision the agreement on the duration of sickness absence was poor, but the agreement regarding the occurrence of sickness absence was good.

## Conclusions

PGP is a frequent and major cause of sick leave in pregnancy. We have retrospectively identified how it affects pregnant women’s ADL, and found unexpected differences in pain appreciation between women on sick leave and not on sick leave during pregnancy. A coping factor seem to be present, most likely dependent on education, associated with work situation and/or work posture, which decreases sick leave. We recommend that these issues should be further examined in a prospective longitudinal study since it may have important implications for sick leave frequency during pregnancy.
